# Preoperative pulmonary nodule localization: A comparison of hook wire and Lung‐pro‐guided surgical markers

**DOI:** 10.1111/crj.13726

**Published:** 2023-12-20

**Authors:** Rui He, Chao Ming, Yujie Lei, Wanling Chen, Lianhua Ye, Guangjian Li, Xiangwu Zhang, Boyi Jiang, Teng Zeng, Yunchao Huang, Guangqiang Zhao

**Affiliations:** ^1^ Department of Thoracic Surgery I Third Affiliated Hospital of Kunming Medical University (Yunnan Cancer Hospital, Yunnan Cancer Center) Kunming China

**Keywords:** hook‐wire, Lung‐pro, preoperative localization, solitary pulmonary nodules, video‐assisted thoracoscopic surgery

## Abstract

In minimally invasive thoracoscopic surgery, for solitary pulmonary nodules (SPNs) far from the pleura, it is difficult to resected by only relying on imaging data, and effective preoperative localization can significantly improve the success rate of surgery. Therefore, preoperative localization is particularly important for accurate resection. Here, we compare the value of a novel Lung‐pro‐guided localization technique with Hook‐wire localization in video‐assisted thoracoscopic surgery.

**Method:**

In this study, 70 patients who underwent CT‐guided Hook‐wire localization and Lung‐pro guided surgical marker localization before VATS‐based SPNs resection between May 2020 and March 2021 were analyzed, and the clinical efficacy and complication rate of the two groups were compared.

**Result:**

Thirty‐five patients underwent Lung‐pro guided surgical marker localization, and 35 patients underwent CT‐guided Hook‐wire localization. The localization success rates were 94.3% and 88.6%, respectively (*p* = 0.673). Compared with the puncture group, the locating time in the Lung‐pro group was significantly shorter (*p* = 0.000), and the wedge resection time was slightly shorter than that in the puncture group (*P* = 0.035). There were no significant differences in the success rate of localization, localization complications, intraoperative blood loss, postoperative hospital stay, and the number of staplers used.

**Conclusion:**

The above studies show that the Lung‐pro guided surgical marker localization and the CT‐guided Hook‐wire localization have shown good safety and effectiveness. However, the Lung‐pro guided surgical marker localization may show more safety than the Hook‐wire and can improve the patient's perioperative experience.

## Graphical abstract

Lung‐pro guided surgical markers have certain advantages in preoperative pulmonary nodule localization.

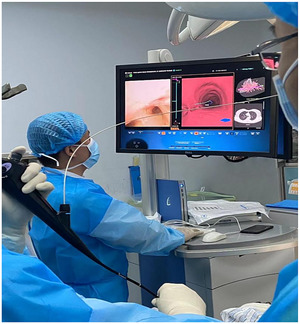

## 1 INTRODUCTION

With the popularity of low‐dose computed tomography (LDCT) scanning, more and more solitary pulmonary nodules (SPNs) are being seen. Most current guidelines recommend tissue biopsy or surgical resection for nodules of ≥8 mm in diameter with a high likelihood of malignancy suggested by enhanced CT and/or PET‐CT.^1^, ^2^ According to Tsukada et al.,^3^ the success rate of percutaneous needle biopsy was 66.7% to 78.9% for SPNs with diameters ranging from 6 mm to 20 mm. With the development of video‐assisted thoracoscopic technology (VATS),^4^ the pattern of diagnosis and treatment of SPNs has been improved. However, for some deep‐seated SPNs, due to visual and tactile limitations of thoracoscopic surgery, intraoperative resection of SPNs is difficult to rely only on the patient's imaging data, and the target lesion is easy to be missed, which leads to prolonged operation time, expanded resection scope, and even unplanned thoracotomy. Intraoperative identification and palpation failures have been reported in 54–63% of patients without preoperative localization.^5^ Therefore, preoperative localization contributes to accurate resection of SPNs. At present, the most widely used localization techniques in clinic include preoperative CT guided localization (coil, Hook‐wire, and medical glue), percutaneous fluid injection (methylene blue), and intraoperative ultrasound (US) and electromagnetic navigation bronchoscopy (ENB) system.^5^, ^6^, ^7^ Hook‐wire is the most widely used in clinical applications due to its high successful rate (80.6–99.6%).^8^ However, when Hook‐wire improves the success rate of our operation, it is also accompanied by adverse complications such as pneumothorax, hemothorax, pleural reaction, and air embolism.^9^ In particular, the pain after localization will lead to patients' anxiety and tension, which will affect patients' experience in perioperative period. Therefore, we propose a novel technology for localization after general anesthesia intubation using Lung‐pro navigation system and evaluate the value of this technology by comparing it with Hook‐wire.

## 2 MATERIALS AND METHODS

### 2.1 Clinical data

Our work is a prospective study that prospectively included 35 patients who underwent lung‐PRO‐guided surgical marker localization at our center between May 2020 and March 2021. We retrospectively included 35 cases of CT‐guided Hook‐wire locating in our center from May 2020 to December 2020 according to 1:1 matching. All cases were unanimously determined by senior thoracic surgeons whether preoperative positioning was necessary. This study has been registered with clinicaltrial.gov, registration number NCT04139408. The study was carried out in accordance with the Declaration of Helsinki. The study protocol and informed consent were approved by the Ethics Committee of the Third Affiliated Hospital of Kunming Medical University (No. YW202019) with written‐informed consent from all participants. (Table 1).

**TABLE 1:** Inclusion and exclusion criteria.

Inclusion criteria	Exclusion criteria
0.5 cm ≤ maximum diameter of lung nodules≤ 2 cm	With local lymph node metastasis or distant metastasis
Lung nodule≥ 5 mm from the visceral pleura	Severe adhesions in the chest cavity interfering with surgical maneuvers
Difficulty in intraoperative nodal localization based on preoperative CT	

### 2.2 Equipment and materials

The equipment and materials used for localization in each group were as follows: Lung‐pro group: Lung‐pro navigational bronchoscopy system (BRONCUS, United States), conventional bronchoscope (4.9 mm outer diameter, Olympus, Japan), disposable pulmonary surgical markers (HM‐SD, Hangzhou Broncus Medical Co., Ltd.); puncture group: Hook‐wire (Accura TM BLN, 21G [0.8 mm] × 100 mm, ARGON, USA), CT (64‐row spiral CT, Philips).

### 2.3 Localization method

#### 2.3.1 Lung‐pro group

**FIGURE 1:**
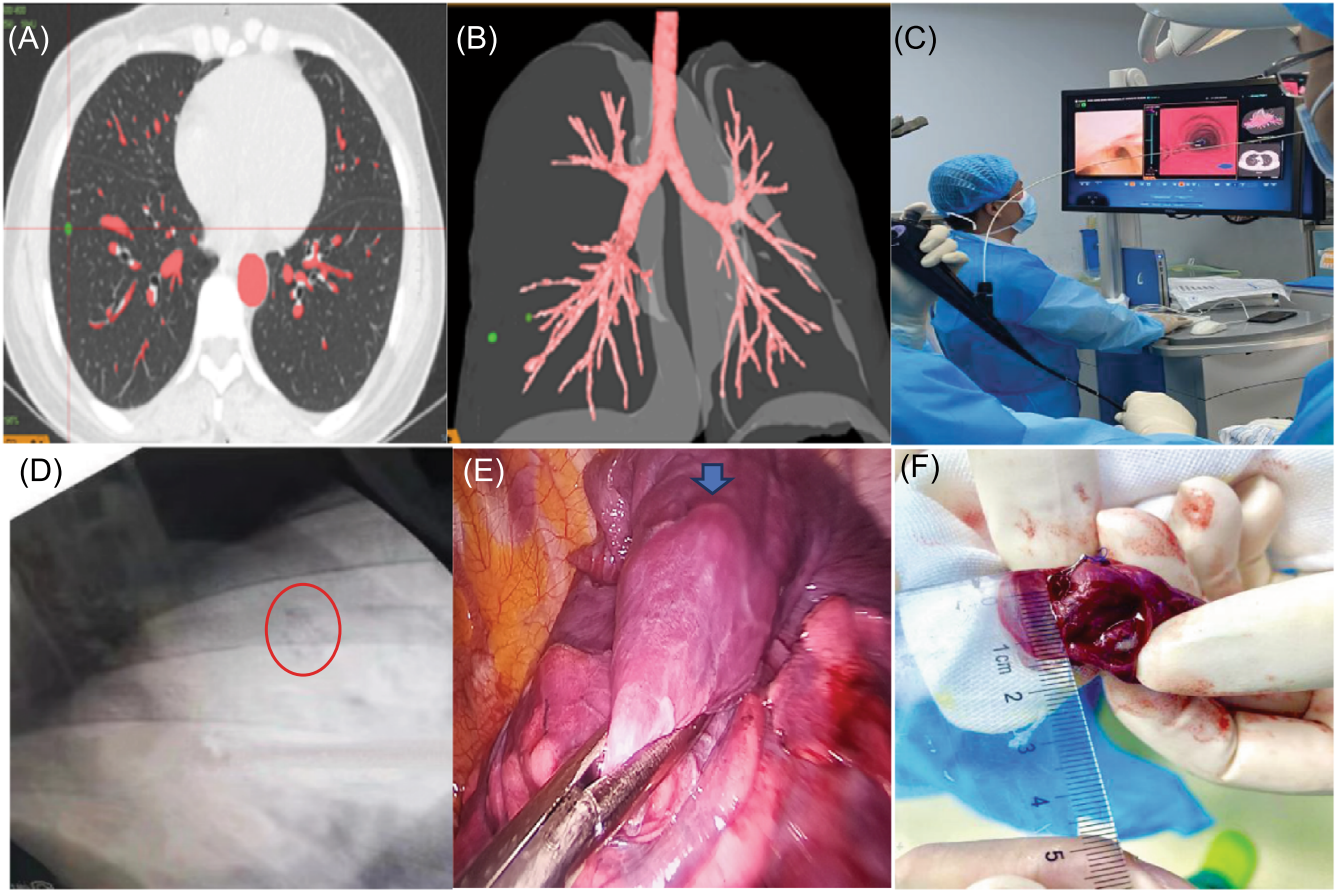
The following photos are from the Third Affiliated Hospital of Kunming Medical University: (A) preoperative CT images, (B) 3D model of Lung‐pro reconstruction; (C) marker placement procedure, (D) DR confirms surgical marker location; (E) markers seen intraoperatively (at arrows); (F) resected markers and nodules.

The preoperative Lung‐pro bronchoscopic navigation system reconstructed the HRCT data of the patient into a virtual three‐dimensional model (Figure 1A,B), and planned the optimal path according to the location of the target nodule. After entering the patient's airway through the electronic bronchoscope, the real‐time image was matched with the virtual 3D model image (Figure 1C), and the bronchoscope was guided to the target localization according to the navigation path established before surgery. The transmitter was reached to the farthest end of the bronchoscope along the bronchoscope channel, and the marker was released when the distance between the distal sheath tube of the transmitter and the target localization was less than 1 cm. The direction and distance between the marker and the lesion were determined according to the Lung‐pro 3D model, and the location of the marker was determined with digit radiography (DR) (Figure 1D). Then, the lateral localization was taken, and a bronchial occluder was inserted into the single cavity trachea catheter to achieve single lung ventilation, and a single‐hole thoracoscopic pulmonary wedge resection was performed (Figure 1E).

#### 2.3.2 Puncture group

Patients underwent CT‐guided Hook‐wire localization before anesthesia. Before localization, the size of the nodule, the distance from the pleura, and the relationship between the surrounding tissues were clarified according to HRCT. The puncture localization, needle path, depth, and angle were determined beforehand. After routine disinfection and toweling, 2% lidocaine local infiltration anesthesia was applied, and the localization needle cannula was passed through the lung tissue under CT guidance according to the previously designed needle path, and the Hook‐wire needle was released and recovered after reaching the predetermined depth, so as to determine the localization of the Hook‐wire needle in relation to the target nodule by CT. The extracorporeal guidewire was left in place for an appropriate length and fixed to the body surface, and then, a double‐lumen endotracheal tube was inserted under general intravenous anesthesia, and single‐hole thoracoscopic pulmonary wedge resection was performed, which had to be performed within 2 h after localization, and during which the patient was observed for symptoms such as chest pain, chest tightness, and dyspnea.

### 2.4 Surgical method

All patients were treated under general intravenous anesthesia by single‐or double‐lumen tracheal intubation in a single lung. A 3–5 cm incision was made in the fourth or fifth intercostal space of the midaxillary line. In the Lung‐pro group, after Lung collapse, there was usually an obvious protrusion at the location of the marker, so as to determine the location of the marker, and to further confirm the location of the marker by sliding the finger or the instrument. In the puncture group, the guide wire was first detected for shedding and displacement. If there was no shedding or displacement, pulmonary wedge resection was performed according to the localization of the Hook‐wire needle, and the cutting edge was ensured to be above 2 cm. After removing the removed lung tissue, the surface is dissected with a scalpel, looking for nodules, and the distance from the surgical marker or Hook‐wire needle to the nodules is measured (Figure 1F). In addition, rapid pathologic examination was performed for the resected lesion. When invasive lung cancer was identified, lobectomy or segmentectomy and systematic lymph node dissection were performed.

### 2.5 Data collection and statistical methods

Dates on gender, age, nodule location, nodule size, nodule distance from pleura, pathologic type, and previous tumor history of patients in the two groups were collected, and the locating time, locating success rate, pulmonary wedge resection time, introperative number of staplers used, introperative blood loss, location‐related complications, postoperative complications, and postoperative hospitalization time of each case were analyzed.

For the Lung‐pro group, resection of the marker and the target pulmonary nodule by VATS surgery, and the nearest distance of the marker from the edge of the pulmonary nodule not more than 2 cm were considered successful localization; the time from bronchoscope insertion into the airway to bronchoscope withdrawal was defined as the localization time. For the puncture group, successful localization was regarded as no intraoperative guidewire dislocation or displacement, and successful resection of the lesion according to the localization of the localized guidewire, with the closest distance of the Hook‐wire needle from the edge of the lung nodule ≤2 cm; the time from the first CT scan to the fixation of the wire ex vivo was defined as the time of localization. Lung wedge resection time was defined as the length of time from the establishment of the VATS channel establishment to the dissection of the specimen to find the nodule.

The above data were analyzed using SPSS 21.0 statistical software. The results were expressed as Mean ± SD, the comparison of means between multiple groups was analyzed by ANOVA, and *p* < 0.05 was regarded as a statistically significant difference.

## 3 RESULT

### 3.1 Patients characteristics

All patients successfully completed the operation without perioperative death. There were no statistically significant differences between the two groups in age, gender, previous tumor history, nodule location, nodule diameter, nodule distance from pleura, and postoperative pathological properties (Table 2).

**TABLE 2:** General information of patients in both groups.

	Lung‐pro group	Puncture group	t/*χ* ^2^	*P* value
Age (years)	51.97 ± 9.95	51.29 ± 7.28	0.329	0.743
Gender			0.60	0.806
Male	13	14		
Female	22	21		
History of malignant tumors			0.215	0.643
Yes	3	2		
No	33	33		
Diameter (mm)	0.87 ± 0.28	0.92 ± 0.29	−0.962	0.339
Distance from pleura (mm)	1.60 ± 0.60	1.59 ± 0.53	0.084	0.933
Position			2.375	0.667
Upper lobe of the right lung	8	11		
Middle lobe of the right lung	1	1		
Lower lobe of the right lung	15	11		
Upper lobe of the left lung	3	6		
Lower lobe of the left lung	8	6		
Postoperative pathology			1.433	0.231
Benign (e.g., tumor)	21	16		
Mesothelioma	14	19		

### 3.2 Intraoperative and postoperative observations

Among 35 patients who underwent Lung‐pro guided surgical marker localization, 33 cases were successfully located, and 2 cases were failed, with a success rate of 94.2%. The reason for failure was that the distance between the marker and the nodule was >2 cm after Lung wedge resection (2.2 and 2.3 cm, respectively), but all patients were successfully resected, and the target nodule was found. Among the 35 patients who underwent Hook‐wire puncture localization guided by CT, 31 cases were successfully located, and 4 cases failed, with a success rate of 88.6%. The reason for the failure was that the localization hook was found to have fallen off during intraoperative exploration. Among the 35 patients, nodules were found and successfully resection according to the pulmonary parenchymosis after the removal of the localization hook. In two patients, the surgeon determined the location of the nodules according to preoperative CT and successfully removed the nodules. In two patients, a small amount of hemostasis was found in the chest during thoracic exploration. One patient in the Lung‐pro group had persistent air leakage for more than 5 days after surgery, and two patients in the puncture group had postoperative Lung infection. The above complications were considered to be independent of location, and all patients were discharged successfully after treatment.

Compared with the puncture group, the localization time of Lung‐pro group was shorter (10.9 min vs. 20.2 min), and the wedge resection time of lesion was shorter (16.4 min vs. 20.4 min), and the differences were statistically significant. There was no significant difference between the two groups in the success rate of localization, intraoperative blood loss, intraoperative nail bin use, postoperative hospital stay, and postoperative complications (Table 3).

**TABLE 3:** Relevant intraoperative and postoperative data of two groups of patients.

	Lung‐pro group	Puncture group	t/*χ* ^2^	*P* value
Positioning success rate%	94.3%	88.6%	0.729	0.673
Positioning time (min)	10.9 ± 8.5	20.2 ± 4.4	−5.791	0.000
Wedge resection time (min)	16.4 ± 8.9	20.4 ± 6.6	−2.145	0.035
Intraoperative bleeding volume (ml)	88.57 ± 69.75	73.71 ± 42.91	1.073	0.287
Length of postoperative hospitalization (days)	3.7 ± 0.7	3.5 ± 0.6	1.396	0.167
Number of nail bins in use (pcs)	3.94 ± 1.86	3.37 ± 0.77	1.678	0.098

### 3.3 Localization‐related complications

In the puncture group, 22 patients complained of mild pain while waiting for surgery, but no intervention was performed. Pain was a subjective feeling after patient localization, and we did not include pain in the complications related to localization. In addition, pneumothorax occurred in two patients, puncture localization‐related bleeding was observed in two patients during the operation, and no hemoptysis, pleural reaction, air embolism, and other complications occurred; the complication rate was 11.4%. Since all patients in the Lung‐pro group were operated under general anesthesia, no subjective complications (such as pain) occurred, and no location‐related complications such as bleeding were found during the operation (Table 4).

**TABLE 4:** Localization related complications in puncture group.

Complications	Pneumothorax	Bleed	No. of complications
Frequency	2	2	31
Rate%	5.7%	5.7%	88.6%

## 4 DISCUSSION

In recent years, with the popularization of “early diagnosis and early treatment” of lung cancer, more and more SPNs have been detected, while conventional examination methods are difficult to determine the pathological diagnosis of SPN, and diagnostic surgery with VATS has become the first choice for the diagnosis and treatment of SPNs.^10^ Under the premise of conforming to the concept of minimally invasive, VATS technology can not only reduce the pain of patients but also shorten the time of patients with tubes and hospitalization. However, due to the reduction of the incision, the surgeon is limited to directly touch the SPNs, resulting in part of the SPNs cannot be accurately and quickly found and excised, therefore, increasing the operation difficulty of the surgeon, but also restricting the development of VATS technology. A review by Zaman et al.^11^ suggests that finger palpation is less effective and should be avoided as much as possible. In order to solve this problem, many localization techniques for SPNs have appeared at home and abroad to achieve accurate localization and excision of lesions. Common techniques include CT‐guided percutaneous localization, intraoperative ultrasound localization, and ENB dye localization.^12^ At present, the most common localization method used in percutaneous localization is Hook‐wire localization.^13^ The most common complications of this technique are pneumothorax, with an incidence rate of 7.5–40%,^14^ and displacement rate of 2.5–13%.^15^ Wang et al.^16^ report a novel claw‐shaped localization needle with a shift rate of only 0.5%. ENB‐guided dye marking is increasingly favored by thoracic surgeons. Like this study, ENB dye marking is also carried out under general anesthesia to avoid discomfort after patient localization. However, ENB dye marking is the same as many dye localization (such as methylene blue), and it is sometimes difficult to determine the target area for some patients with more pigmentation on the lung surface. Song et al.^17^ analyzed the results of ENB dye marking localization in 94 patients with SPNs, and the results showed that the accuracy was comparable to that of Hook‐wire localization, and no ENB‐related localization complications were observed. A meta‐analysis showed that ENB biopsy with controlled ventilation using general anesthesia had better diagnostic rates than uncontrolled respiratory movement, an observation that suggests the accuracy of ENB localization is limited by respiratory movement.^18^ At the same time, delayed operation after ENB‐guided dye marking also increases the risk of complications.^18^ Ultrasound localization technology has also brought excellent results in the localization of SPNs, but because air has a certain obstruction effect on ultrasound, it is difficult to locate patients with emphysema.^19^

In this study, 35 patients who underwent CT‐guided Hook‐wire localization were strictly required to undergo surgery within 2 h after localization, but there were still four patients (11.4%) with guide wire falling off during the operation, which was close to the rate of 2.5–13% reported by Zhao et al.^15^ We analyzed the main reasons for the loss of guide wire as follows: (1) In the process of transport, such as pain, cough, localization change and other reasons can lead to the loss of guide wire; (2) relevant studies have reported that for some shallow nodules, the decoupling rate may increase,^9^ because the tip part of the localization needle is not fully opened, resulting in the puncture needle slipping without anchoring the target. Among the 35 patients who received Lung‐pro navigation localization, 33 patients were successfully localization, and the localization success rate was 94.3%. The reason for the failure was that the distance from the lesion to the marker was >2 cm. However, all patients successfully completed the operation and successfully found the nodule. The main reasons for the failure of our analysis are as follows: (1) The Lung‐pro navigation system is a 3D virtual model automatically generated according to the 2D CT data of the patient. Without further optimization on the original 3D image in the later stage, there may be certain errors between the virtual image and the patient's own anatomical structure; (2) the technology requires the operator to operate in a small space, and the technical level of the operator is high, so the level of the operator may also lead to a slight shift of the release marker; (3) during thoracoscopic surgery, the finger or instrument sliding palpation on the surface of the lung tissue may cause compression of the lung tissue, resulting in the displacement of the disposable markers. Based on the above inference, we believe that the key to improve the success rate of Hook‐wire puncture localization under CT guidance is to stabilize the guide wire and prevent it from falling off. In fact, Iguchi et al.^20^ have reported that by using nylon sutures to fix Hook‐wire, the dislocation rate can be reduced to 0.4–2.5%. For Lung‐pro guided surgical marker localization, more skilled localization techniques and more gentle intraoperative sliding palpation may affect the localization success rate.

In terms of localization time and operation time, Lung‐pro group was superior to puncture group in this study. In terms of localization time, the two localization methods have different operating processes, operators, and their respective advantages, and the final difference is only 10 min (10.9 min vs. 20.2 min), which has little impact on the overall operation time, so there is no strong reference significance. We tend to interpret the result as that Lung‐pro navigation, and localization technology is also a fast localization method, but the specific localization method should be determined according to the factors of each hospital, physician, and patient, and it is not necessary to focus on the difference in operation time between the two localization methods. In terms of operation time, all cases included in this study were performed by a team with skilled operation experience in the Thoracic Surgery Department of the Third Affiliated Hospital of Kunming Medical University. The wedge‐shaped resection time of the Lung‐pro group was faster than that of the puncture group (16.4 min vs. 20.4 min). We believe that this result was caused by the following two reasons: First, Hook‐wire localization requires treatment of Hook‐wire needles before performing Lung nodule wedge‐shaped resection, while Lung‐pro navigational localization surgery only requires palpation markers at the protrusion and then lung wedge‐shaped resection; second, it is difficult for Hook‐wire localization to intuitively determine the depth of nodules from visceral pleura through intraoperative palpation, while the palpation feel of Lung‐pro navigation localization is closer to that of traditional non‐localized surgery. Based on the above two points, we believe that Lung‐pro navigation‐oriented surgery has fewer procedures and is more similar to traditional non‐localization surgery, which is more suitable for young doctors with less experience to learn. In addition, we considered defining the localization time of patients as the duration of general anesthesia, but in fact, we found during the operation that the Lung‐pro group occupied part of the anesthesia time during the localization process, although this time was only 10.9 min, it still affected the objectivity of the duration of anesthesia. At the same time, both groups of patients had extensive resection due to the pathological type of invasive adenocarcinoma, so we did not further compare the duration of general anesthesia between the two groups.

In terms of location‐related complications, two cases of hemorrhage and two cases of pneumothorax were found in the puncture group in this study, and no serious complications such as hemothorax and air embolism were found. The overall complication rate was 11.4%. Lower than 22% reported by Park et al.^21^ in the meta‐analysis, our analysis may be caused by the small sample size in this study, as well as the influence of nodule depth and location. No significant location‐related complications were found in the Lung‐pro group, which may be related to the transition to surgery immediately after localization. It is worth mentioning that 74.3% of patients experienced mild pain after CT‐guided Hook‐wire puncture localization. However, this pain was believed to be based on patients' subjective consciousness and did not seem to affect the accuracy of localization in this study. Therefore, we did not consider pain as a complication related to localization. However, we believe that the more intense pain may limit the patient's breathing movement, which may increase the risk of displacement of the localization needle. However, Lung‐pro navigation and localization are performed after the patient's general anesthesia. Although the results show that the incidence of complications related to the localization of Lung‐pro is not significantly different from that of the puncture group, it can greatly avoid the pain of patients waiting for surgery, as well as the resulting psychological stress and fear, so as to improve the experience of patients in the perioperative period.

Lung‐pro navigation system is a virtual bronchoscopy technology based on CT images. Compared with traditional CT‐guided percutaneous puncture localization technology, the most significant advantage of this technology is that the release of markers is carried out in the same environment under general anesthesia, avoiding the subjective discomfort of patients. It can also achieve the purpose of full control of ventilation, low invasiveness, and high accuracy, and the transition to thoracoscopic surgery immediately after localization can reduce the adverse reactions of related complications and timely management. In addition, the surgical markers used in this study benefit from their self‐dilatability and spindle design, which avoids the risk of vascular injury easily caused by traditional hook‐shaped localization needles and is more conducive to the exploration and perception of tiny nodules during surgery.

At present, the widely used localization technology in clinical practice inevitably needs to be exposed to radiation from fluoroscopy equipment, and this study is also the same. After the localization is completed, we need to confirm the marker by DR. Because our study is in the stage of a single‐stage clinical experiment, in the event that the safety and efficacy of this study are validated in future multicenter prospective studies, we may be able to dispense with this step, which would be beneficial to both patients and healthcare providers.

It is worth mentioning that for some nodules far from the pleura, we usually need to release the locator at a deeper position to get as close as possible to the target nodules to improve the localization success rate. Therefore, the problem of locator residue may occur in many solid locators. Although we have some reference to the spatial position of markers and nodules according to the virtual image of the patient during the operation, it is undeniable that the residual problem of the locator is still inevitable in the future work. Moreover, we believe that in this study, surgical markers seem to be more likely to have marker locator residue than Hook‐wire, which may be the same as the surgical markers we used were completely located in the lung tissue, and the operation of surgical instruments may cause the markers to shift during the marking resection range, increasing the potential possibility of marker residue. The widely used percutaneous puncture needle usually has a tail line and is fixed on the body surface. During surgery, after fixing the excision area, the tail line can be pulled to ensure the integrity of the locating needle and prevent it from remaining in the lung tissue. At the same time, a locator with a tail line device can be used as traction during surgery, and as early as 1992, Plunkett et al.^22^ proposed that the anchoring force of the locator needle could act as a handle on which the surgeon could apply traction to help remove deep lung lesions. Once the locator or lesion remains, it is very difficult to perform an extended excision, and blind excision may distort the lung lobes, leading to adverse events such as atelectasis, which would be disastrous for most thoracic surgeons.

In summary, compared with Hook‐wire puncture localization, Lung‐pro navigation localization shows equivalence in localization success rate, number of nail bin used, intraoperative blood loss, postoperative hospital stay, and other indicators, and its localization safety is more, which can effectively improve the perioperative experience of patients. For the surgeon, the surgical process is less, more similar to the traditional non‐localization surgery, easy for surgeons to learn and master. However, there are some limitations to this study, some of which are prospective and some of which are retrospective, and the effectiveness of localization needs to be concluded by multi‐center, prospective, and larger sample clinical studies. In addition, as a new technology, Lung‐pro navigation and localization require the operator to accumulate experience to improve the success rate of localization. The limitation lies in the high technical level and equipment requirements for operators, which limits its promotion in grass‐roots hospitals.

## AUTHOR CONTRIBUTIONS

*Conception and design*: Rui He and Chao Ming. *Administrative support*: Yunchao Huang. *Provision of study materials or patients*: Teng Zeng and Boyi Jinag. *Collection and assembly of data*: Rui He. *Data analysis and interpretation*: Rui He, Chao Ming, and Xiangwu Zhang. *Manuscript writing*: Rui He. *Final approval of manuscript*: Guangqiang Zhao, Yunchao Huang, Yujie Lei, Lianhua Ye, Wanlin Chen, and Guangjian Li.

## CONFLICT OF INTEREST STATEMENT

All authors declare that there is no conflict of interest.

## ETHICS STATEMENT

The authors are accountable for all aspects of the work in ensuring that questions related to the accuracy or integrity of any part of the work are appropriately investigated and resolved. The study was conducted in accordance with the Declaration of Helsinki (as revised in 2013). The study was approved by the Institutional Review Board of the Third Affiliated Hospital of Kunming Medical University (No. YW202019). The clinical trial registration number is NCT04139408 (clinicaltrial.gov), and all patients signed preoperative informed consent for surgery.

## Data Availability

The data that support the findings of this study are openly available upon request.
